# [Retracted] Upregulation of estrogen receptor mediates migration, invasion and proliferation of endometrial carcinoma cells by regulating the PI3K/AKT/mTOR pathway

**DOI:** 10.3892/or.2024.8856

**Published:** 2024-12-16

**Authors:** Xinxin Hou, Meng Zhao, Tong Wang, Guiyu Zhang

Oncol Rep 31: 1175–1182, 2014; DOI: 10.3892/or.2013.2944

Following the publication of the above article, a concerned reader drew to the Editor's attention that a couple of the figures in this paper contained overlapping sections, where data which were intended to show the results from differently performed experiments had been derived from the same original source. First, regarding the cell transfection experiments in Fig. 1, the Con, ERα and ERβ data panels in Fig. 1A all contained overlapping sections of data; secondly, comparing the data panels for the cell migration and invasion assays shown in Fig. 5, three pairs of panels were also found to contain overlapping data.

In view of the fact that these identifications were made in terms of overlapping data panels, the Editor of *Oncology Reports* has decided that this paper should be retracted from the Journal on account of a lack of confidence in the presented data. The authors were asked for an explanation to account for these concerns, but the Editorial Office did not receive a satisfactory reply. The Editor apologizes to the readership for any inconvenience caused.

